# Endoscopic incisional therapy for esophageal strictures in children: a retrospective cohort study

**DOI:** 10.1007/s00464-025-12477-8

**Published:** 2025-12-20

**Authors:** Or Genzer Rechtman, Mordechai Slae

**Affiliations:** 1 Department of the Pediatric Gastroenterology, Hadassah-Hebrew University Medical Center, POB 12000, 91120 Jerusalem, Israel; 2 Faculty of Medicine, Hebrew University of Jerusalem, Jerusalem, Israel

**Keywords:** Esophageal stenosis, Electrosurgery, Surgical endoscopy, Pediatrics, Minimally invasive surgical procedures

## Abstract

**Background and Aims:**

Pediatric esophageal strictures present a significant clinical challenge. Recently, endoscopic incisional therapy has gained attention as a novel treatment approach. This study aims to assess the efficacy and safety of this procedure in managing pediatric esophageal strictures and to identify factors that influence treatment outcomes.

**Methods:**

This retrospective cohort study analyzed endoscopic incisional therapy procedures performed on pediatric patients at a tertiary care center between November 2019 and May 2023. Patient demographics, stricture characteristics, procedural details, and clinical outcomes were collected. Treatment success was defined as complete symptom resolution without further intervention for at least three months, while improvement indicated symptom reduction requiring additional treatment, and failure necessitated surgery. Fisher’s exact and Wilcoxon-Mann-Whitney tests were used to assess associations between variables and outcomes.

**Results:**

The cohort consisted of 22 pediatric patients (mean age 6.6 ± 6.1 years; 41% female) who underwent a total of 101 procedures, including balloon dilation in 63 cases. Stricture etiologies included tracheoesophageal fistula (50%), caustic ingestion (27%) and others. Complete symptom resolution occurred in 68% of patients, while 23% showed improvement, and 9% required surgery. No significant associations were found between outcome and stricture location, severity, etiology, or medications. However, multiple strictures (*p* = 0.005) and shorter intervals between procedures (*p* = 0.02) were associated with reduced success. No procedure-related complications were observed.

**Conclusions:**

Endoscopic incisional therapy is a safe and effective treatment for pediatric esophageal strictures, achieving symptom resolution in most cases. Further studies are needed to optimize patient selection and refine treatment strategies.

**Supplementary Information:**

The online version contains supplementary material available at 10.1007/s00464-025-12477-8.

Pathological narrowing of the esophageal lumen, known as esophageal strictures, presents a significant impairment to its functionality. In pediatric cases, these strictures are predominantly benign and arise from a variety of causes [1]. These include acquired factors such as ingestion of caustic substances or disk batteries, gastroesophageal reflux disease (GERD), eosinophilic esophagitis (EoE), exposure to radiation therapy, and graft versus host disease [1]. In children, the most prevalent congenital cause of esophageal stricture is surgical repair of esophageal atresia (EA) [2–4]. Other congenital causes, though less common, include epithelial disorders such as epidermolysis bullosa [5]. Diagnostic approaches typically involve upper endoscopy or esophagography [6]. Treatment primarily aims at alleviating symptoms like dysphagia, drooling, coughing, food impaction, regurgitation, and failure to thrive [7]. However, achieving complete recovery of the stricture area is often challenging [8]. Consequently, multiple dilatation sessions are generally necessary to achieve the therapeutic objective [8].

Balloon dilatation is a widely accepted technique for addressing esophageal strictures [1]. This method employs balloon dilators to exert even radial forces throughout the stricture area [1]. At present, Through-the-Scope (TTS) balloon dilators are the most favored variant, offering the advantage of dilating the stricture under direct visual observation using an endoscope [9, 10]. The pediatric literature reviewing this method has been retrospective. While certain studies demonstrate long-term efficacy, others have only indicated short-term effectiveness [11, 12].

Endoscopic incisional therapy (EIT) is a minimally invasive procedure employed in the management of esophageal strictures. This technique utilizes an endoscopic knife to create incisions through the stricture, employing monopolar high-frequency electrosurgery. The electrosurgical application involves a dual-phase action, commencing with a cutting cycle for incision, followed by a coagulation cycle to achieve hemostasis [13]. The use of EIT in pediatric esophageal strictures has been documented in only a few reports [14, 15]. Manfredi et al. described the outcomes of 133 esophageal EIT procedures in pediatric patients, reporting a success rate of 61% to 100% (for refractory and non-refractory strictures, respectively) and an adverse event rate of 5.3%. Tan et al. reported on seven pediatric patients undergoing esophageal EIT (with additional stenting in some) all of whom achieved remission of symptoms with no significant complications. Two other single cases have been reported [16, 17]. This study primarily aims to evaluate the efficacy and safety of EIT in a pediatric population presenting with esophageal strictures. Secondarily, the study seeks to determine the influence of stricture characteristics, such as anatomical location and etiology, as well as adjunctive therapies including pharmacologic interventions and balloon dilation, on the overall effectiveness and safety profile of EIT.

## Methods

In this retrospective cohort analysis, we examined the efficacy and safety of EIT procedure in pediatric patients treated for esophageal strictures at a tertiary care center. The study was approved and conducted in accordance with the medical organization's ethics committee (reference no. HMO-0505-23).

The study encompassed a review of medical records spanning from November 2019 to May 2023, focusing on pediatric patients who underwent EIT during this period. Various variables were collected: patient variables included age, sex, and the presence of underlying conditions; stricture variables encompassed etiology, esophageal location, and severity; and procedure variables included additional therapies, the total number of EIT sessions, and the time interval between consecutive procedures. Symptomatology was documented both before and after the procedure.

Strictures were classified according to their severity. The categories- mild, moderate, or severe -were based on evaluating four key characteristics: the width and length of the stricture, the extent of dilatation proximal to the stricture, and the number of intra-stricture angles, as detailed in Table 1. The presence of any one of these characteristics was sufficient to categorize the stricture into a specific severity level.

**Table 1:** Definition of severity of the strictures

	Width (mm)	Length (cm)	Proximal dilatation	Intra-stricture angles
Mild	5 or more	Up to 1	None	None
Moderate	3–5	1–2	Present	One
Severe	Up to 3	2 or more	Severe	Two or more

Strictures were classified according to their severity. The categories—mild, moderate, or severe -were based on evaluating four key characteristics: the width and length of the stricture, the extent of dilatation proximal to the stricture, and the number of intra-stricture angles. The presence of any one of these characteristics was sufficient to categorize the stricture into a specific severity level

The safety profile of EIT was assessed through a systematic recording of any complications encountered during and after the procedures.

The EIT was performed using an Olympus pediatric gastroscope. The procedure utilized an electrosurgical unit (ERBE VIO 200s system), an insulated tip (IT) knife (Boston Scientific), with cutting settings adjusted to Endocut I, effect 2, interval 3, and a duration of either 3 or 4.

A single experienced pediatric endoscopist performed all procedures in the endoscopy suite, with patients placed in the supine position and anesthetized under general anesthesia with endotracheal intubation.

Procedure outcome was defined as “success” by post-procedure resolution of symptoms and the absence of a requirement for further medical intervention for at least three months. “Improvement” was defined as a reduction in symptoms, however with the necessity for additional treatment, such as a subsequent procedure. Conversely, “failure” was defined as a scenario necessitating surgical intervention.

To test the association between categorical variables and outcomes, Fisher's Exact Test was used, presenting odds ratios and confidence intervals.

The Wilcoxon-Mann-Whitney test was applied to compare the distribution of the continuous variables across the two outcome categories (Failure or improvement, success). Since certain procedures were pre-planned as part of a sequential series of interventions, and individual procedures could be influenced by prior interventions or external factors, we conducted our analysis on a per-participant rather than a per-procedure basis.

The study was reported in accordance with the STROCSS guidelines for reporting cohort studies in surgery [18].

## Results

The analysis included 101 EIT procedures in 22 children, with 63 of these procedures also involving balloon dilation. The participants had an average age of 6.6 ± 6.1 years at first procedure, with 9 (41%) being female. The defects were primarily located in the middle third of the esophagus (*n *= 13), followed by the upper third (*n* = 8), and the lower third (*n* = 5) (Table 2).

**Table 2:** Patients’ background and procedure outcome

Patient number	Age (years)	Sex	Number of procedures	Location of defect	Etiology	Result
1	13.3	Female	1	Lower third	Caustic ingestion	Success
2	13.2	Female	1	Upper third	TEF	Success
3	13.2	Female	1	Lower third	GERD	Success
4	6.2	Male	1	Upper third, Middle third, Lower third	Dystrophic epidermolysis bullosa	Improvement
5	3.6	Male	2	Middle third	TEF	Success
6	1.4	Male	10	Middle third	TEF	Failure
7	3.7	Male	2	Middle third	Caustic ingestion	Improvement
8	2.4	Male	16	Upper third	Caustic ingestion	Success
9	1.5	Male	2	Middle third	Caustic ingestion	Success
10	2.2	Female	1	Middle third	TEF	Improvement
11	2.6	Male	3	Upper third	TEF	Success
12	1.5	Male	2	Upper third	Dyskeratosis congenita	Success
13	0.9	Male	3	Middle third	TEF	Success
14	1.4	Female	4	Upper third	TEF	Success
15	22.9	Female	3	Upper third	TEF	Success
16	16.3	Male	1	Middle third	GVHD	Success
17	12.1	Female	17*	Upper third, Lower third	Caustic ingestion	Failure
18	10.6	Female	1	Middle third	TEF	Improvement
19	3.2	Male	12	Middle third	TEF	Success
20	2.5	Male	2	Middle third	Idiopathic	Success
21	6.3	Female	8	Middle third	TEF	Success
22	3.3	Male	8	Middle third, Lower third	Caustic ingestion	Improvement

*GERD* gastro-esophageal reflux disease, *GVHD* graft versus host disease, *TEF* tracheo-esophageal fistula. *Paramedical factors influenced the number of procedures

**Fig. 1:**
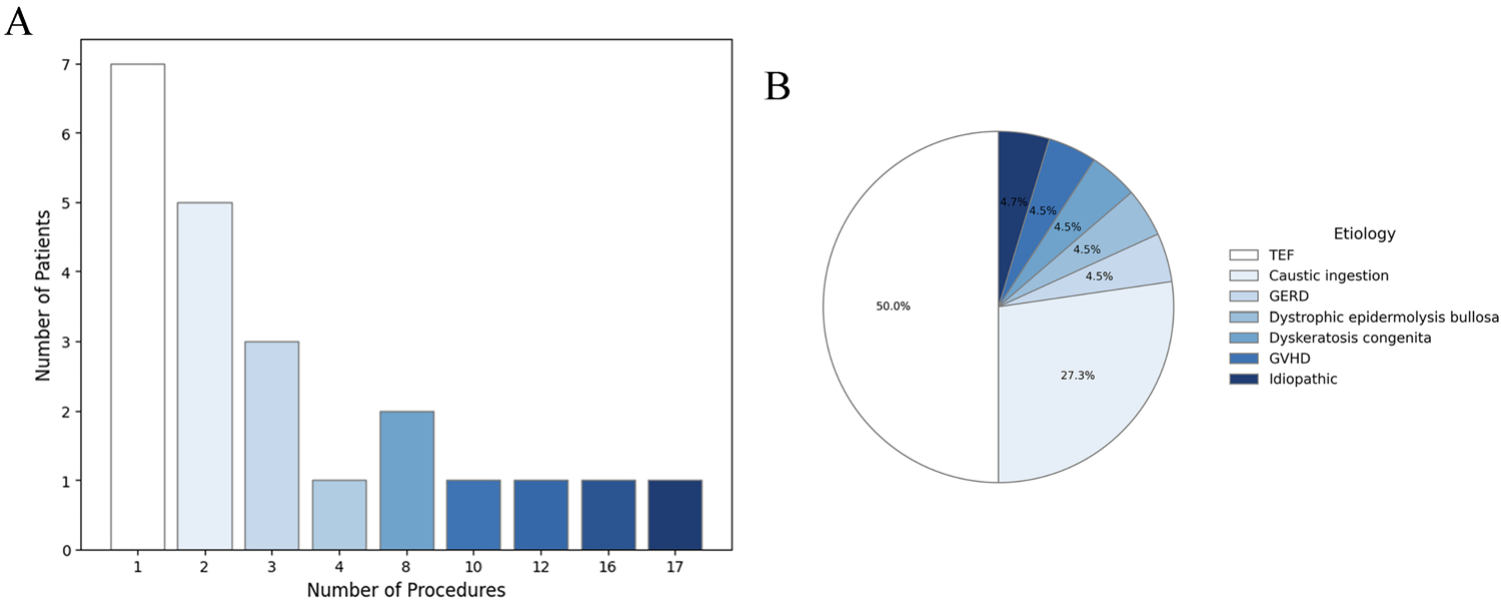
Number of procedures per patient and etiology of esophageal strictures. **A** Among the participants, procedural frequency varied between 1 and 16 procedures. Notably, one participant underwent 17 procedures, however some were dictated by paramedical issues. **B** The causes of strictures varied, including TEF in 11 patient (50%), caustic ingestion in 6 (27%), and other etiologies in the remaining 5 patients (23%). *GERD* gastro-esophageal reflux disease, *GVHD* graft versus host disease, *TEF* tracheo-esophageal fistula

Stricture distribution was as follows: 19 participants had one stricture, 2 had two strictures, and 1 had three strictures (Table 2, Fig. 1A). Seven strictures were classified as mild, 5 as moderate, and 4 as severe. For the remaining participants, there was insufficient information to assess the severity.

The etiologies of the strictures varied, with 11 patients having tracheoesophageal fistula (TEF), 6 patients having caustic ingestion, and the remaining 5 patients having other causes (Fig. 1B).

The mean time between procedures was 104 days (SD = 106 days). Out of the 22 participants, 12 received triamcinolone esophageal injections during the procedures based on clinical judgment, considering expected outcomes or prior medical history. Additionally, some participants were administered topical swallowed budesonide gel either before (*n* = 3) or after (*n* = 7) a procedure, or proton pump inhibitors (PPI) either prior to (*n* = 7) or following (*n* = 8) a procedure.

No instances of perforation occurred during the procedures. However, complications unrelated directly to the procedures were observed, which were associated with anesthesia. These included occurrences of laryngospasm, necessitating early termination of the procedures (grade 1 by Clavien-Dindo Classification).

Since no complications were directly related to the EIT, no risk factors for adverse outcomes could be identified.

**Fig. 2:**
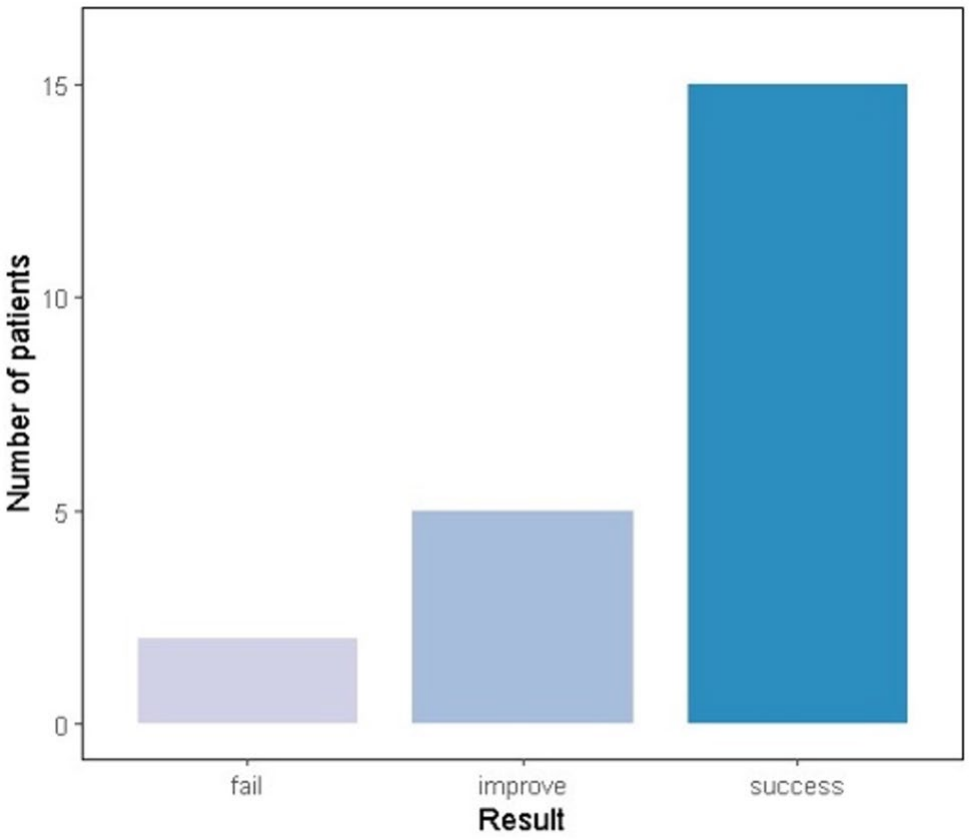
The result of the procedure for each patient. 15 patients achieved successful results with complete resolution of post-procedure symptoms. 5 patients showed improvement and required additional interventions such as balloon dilation. 2 patients failed to respond and needed non-endoscopic surgical intervention. Overall, 20 out of 22 patients (91%) did not require surgery

Fifteen patients (68%) achieved successful outcomes (“Success”) with complete symptom resolution: 4 after one procedure, 4 after two procedures, and the remaining 7 after 3 or more procedures. Another 5 patients (23%) showed partial response but required additional interventions (“Improvement”), including balloon dilations: 3 after one procedure, 1 after two procedures, and the remaining 1 after more than 3 procedures. Despite repeat procedures, two patients (9%) required surgery (“Failure”, Fig. 2). The final analysis, compared the procedures which were categorized as “Success” vs procedures categorized as “Improvement” and “Failure” given that only 2 patients were eventually categorized as “Failure”.

In regard to parameters potentially affecting final patients’ outcome, we tested different patient and procedural parameters. No significant associations were observed between the outcome and the following variables, including patient age at first procedure (median of 3.5, *p* > 0.999) or at last procedure (median of 4.3, *p* = 0. 68), sex (*p* = 0.73), presence of background disease (*p* = 0.27), etiologies of esophageal stricture (*p* = 0.59), number of procedures per patient (*p* = 0.58), defect grade (*p* = 0.67), location of defect (upper third vs. middle or lower third, *p* = 0.26), and medications administered before (oral PPI (*p* = 0.61), oral budesonide gel (*p* = 0.98)), during (triamcinolone intramucosal injection (*p* = 0.73)) or after (oral budesonide gel (p = 0.89), PPI (*p* = 0.49), oral antifungal (*p* = 0.49), oral antibiotics (*p* = 0.35)) the procedure, and the use of a balloon during procedure (*p* = 0.77). The time between procedures varied depending on the outcome, being shorter in the no-success group compared to the success group. Specifically, the minimum time was 11 days vs. 42 days (*p* = 0.05), the maximum time was 46 days vs. 203 days (*p* = 0.09), and the mean time was 28 days vs. 87 days (*p* = 0.02), respectively. Additionally, four patients had more than one stricture (2 or 3). Having multiple strictures was associated with lack of success (*p* = 0.005). Though not statistically significant, TEF was more than twice as prevalent in the success group (73%) compared to the failure group (27%). Caustic injury, on the other hand, was evenly distributed, with 50% in both groups. These parameters are summarized in table number S1.

## Discussion

This study analyzed 101 EIT in 22 children, some of which involved balloon dilation, achieving a complete symptom resolution rate of 68.2%, with some cases necessitating multiple interventions. Predominantly, strictures were located in the middle third of the esophagus and were primarily associated with conditions such as TEF and caustic ingestion. The etiological diversity in our study, with TEF being the most common cause followed by caustic ingestion, emphasizes the variability in stricture pathology that may substantially affect therapeutic outcomes. In our cohort, the association between etiology and outcomes was not significant, however this finding might be a result of small group numbers.

No significant correlations were found between procedural outcomes and variables such as stricture location, severity, etiologies, or medications administered before or after the procedures. These findings suggest EIT success may be influenced by uncaptured factors, such as underlying tissue characteristics, precise procedural techniques, or post-procedural care. This is mostly emphasized by the discorrelation between the parameter of stricture severity and procedural success, as intuitively, short membranous strictures should be more amenable to endoscopic incision than longer, fibrotic strictures. This unexpected finding may reflect that anatomic descriptors do not fully capture the characteristics of an individual patient’s tissue and fibrotic response or stem from the study limitations. Altogether, these findings suggest that EIT outcomes may depend on additional biological or procedural factors not measured in this study. Interestingly, the administration of adjunctive treatments like steroids or mitomycin during procedures, did not significantly influence outcomes, contrasting with previous studies that suggested potential benefits in reducing stricture recurrence [19]. This discrepancy may be attributed to a confluence of factors: the study's limited sample, the heterogeneity of stricture, and the prevalence of challenging cases within the cohort, many of whom had previously experienced failure with conventional balloon dilation. Conversely, the presence of multiple strictures was associated with unsuccessful outcomes, possibly indicating a more severe underlying injury. Similarly, the time between procedures was shorter in the no-success group, perhaps due to more severe strictures. ⁠A key strength of EIT in treating pediatric esophageal strictures is its excellent safety profile; no procedure-related complications or perforations were observed in this study. Furthermore, EIT achieved complete symptom resolution in 68.18% of cases, consistent with previous reports [14, 15]. While 22.73% of patients had partial improvement and 9.09% required surgery despite multiple EIT sessions, these results highlight the need for ongoing research to optimize patient selection and treatment protocols.

**Fig. 3:**
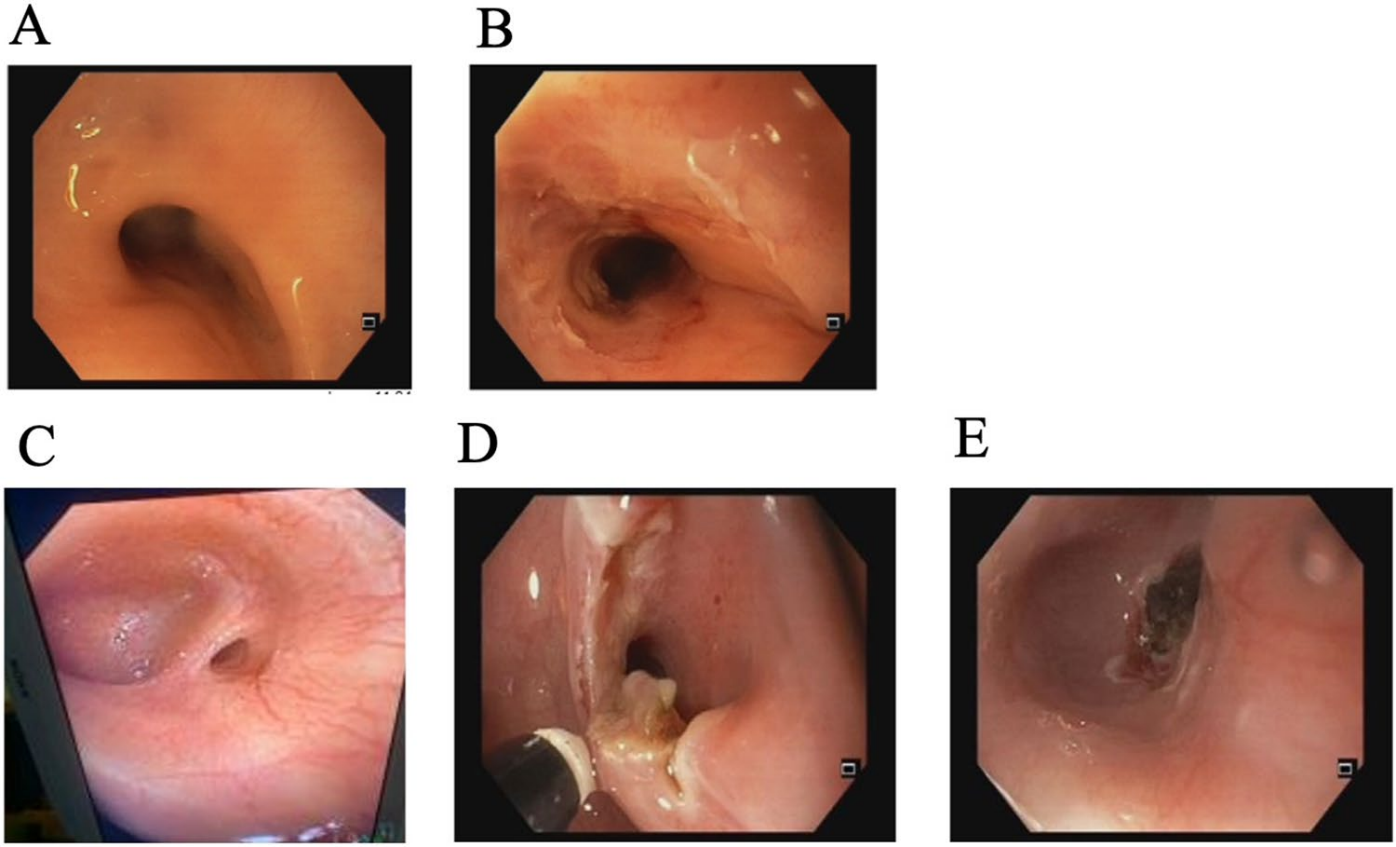
Advantages of Endoscopic Incisional Therapy in complex cases. Asymmetric esophageal stricture before (**A**) and after (**B**) the procedure, resection of pre-stenosis diverticulum (**C–E**)

The procedure demonstrated expansion capabilities comparable to those of a balloon [20] and exhibited potential advantages. In individual cases, the procedure seemed technically more appropriate than a balloon. For example, it was particularly advantageous for treating asymmetric strictures, extremely narrow strictures, or strictures with severe pre-stenosis dilations (Fig. 3). Of note, although not traditionally classified as esophageal strictures, this technique was also employed in the resection of symptomatic esophageal mucosal bridges, the removal of surgical stitches, and other related applications.

This study has several limitations. As a pilot study with a small sample size and a heterogeneous mix of etiologies, the findings should be interpreted with caution, particularly regarding etiology-specific outcomes. Larger cohorts in future studies may help further clarify these potential differences. In addition, the use of several adjunctive therapies alongside EIT may introduce potential confounders, making it harder to fully separate the specific effect of the procedure. Given that this pilot study primarily aimed to assess feasibility and safety, future work with a more uniform treatment approach and an appropriate comparison group could help further clarify the efficacy of EIT.

Finally, the relatively short follow-up period limits our understanding of long-term durability, underscoring the value of extended prospective follow-up in future. Despite these limitations, this report describes a relatively novel procedure, offering valuable early insight into its potential role in the management of pediatric esophageal strictures.

## Conclusions

Esophageal dilation using EIT is proving to be a significant and safe advancement for children with esophageal strictures. While showing promising results, further research is needed to determine which patients will benefit the most from this approach and to maximize its long-term efficacy.

## Supplementary Information

Below is the link to the electronic supplementary material.

Supplementary file1 (PDF 122 KB)

Supplementary file2 (MP4 3815 KB)
