# Supplementary material for: Endoscopic incisional therapy for esophageal strictures in children: a retrospective cohort study
Source: Surg Endosc. 2025 Dec 20;40(1):824–30. doi: 10.1007/s00464-025-12477-8 (PMC12823621; doi:10.1007/s00464-025-12477-8)
Supplement: Supplementary file 1 — Supplementary file1 (PDF 122 KB) [file 464_2025_12477_MOESM1_ESM.pdf]

Table S1 - Correlation of patient and procedural parameters to EIT outcome

	N	Result		Odds Ratio	P value
		Fail or Improve	Success	Success vs Fail or Improve	
**Age at First Appointment**		3.72	3.25		>0.9999
Median[Min,Max]		[1.43,12.07]	[0.91,22.94]		
N	22	7	15		
**Age at Last Appointment**		3.8	4.71		0.6796
Median[Min,Max]		[2.12,13.26]	[1.55,23.24]		
N	22	7	15		
**Gender**					0.7245
Male (%)n	13	4(31%)	9(69%)	reference	
Female (%)n	9	3(33%)	6(67%)	0.9[0.1,8.4]	
**Background Diseases**					0.2678
No (%)n	12	5(42%)	7(58%)	reference	
Yes (%)n	10	2(20%)	8(80%)	2.7[0.3,37.4]	
**Etiology**					0.5937
TEF (%)n	11	3(27%)	8(73%)	reference	
caustic ingestion (%)n	6	3(50%)	3(50%)	0.4[0.0,4.8]	
Other (%)n	5	1(20%)	4(80%)	1.5[0.1,98.2]	
**Number of Procedures**					0.5756
Median[Min,Max]		1.5[1.0,10.0]	2.0[1.0,16.0]		
N	21	6	15		
**Defect Grade**					0.6474
mild (%)n	7	2(29%)	5(71%)	reference	
moderate (%)n	5	1(20%)	4(80%)	1.5[0.1,117.7]	
severe (%)n	4	2(50%)	2(50%)	0.4[0.0,10.1]	
**Location of Defect**					0.2554
(%)upper third n	6	0 0%	6 -100%	reference	
(%)Middle or lower third n	13	4	9	0[0,3.2]	

		-31%	-69%		
**Number of Strictures**					0.0048
1<br>(%)n	18	3<br>-17%	15<br>-83%		
to 3 2<br>(%)n	4	4<br>-100%	0<br>0%		
**Use of triamcinolone injection at the procedure**					0.7322
No<br>(%)n	10	3(30%)	7(70%)	reference	
Yes<br>(%)n	12	4(33%)	8(67%)	0.9[0.1,7.3]	
**Use of PPI medicines before procedure**					0.6129
No<br>(%)n	15	5<br>-33%	10<br>-67%	reference	
Yes<br>(%)n	7	2<br>-29%	5<br>-71%	1.2[0.1,17.4]	
**Use of topical budesonide before procedure**					0.9773
No<br>(%)n	19	5<br>-26%	14<br>-74%	reference	
Yes<br>(%)n	3	2<br>-67%	1<br>-33%	0.2[0.0,4.5]	
**Use of PPI medicines after procedure**					0.4897
No<br>(%)n	14	5(36%)	9(64%)	reference	
Yes<br>(%)n	8	2(25%)	6(75%)	1.6[0.2,22.5]	
**Use of topical budesonide after procedure**					0.8931
No<br>(%)n	15	4<br>-27%	11<br>-73%	reference	
Yes<br>(%)n	7	3<br>-43%	4<br>-57%	0.5[0.1,5.0]	
**Use of Anti-fungal medicines after procedure**					0.4897
No<br>(%)n	14	5(36%)	9(64%)	reference	
Yes<br>(%)n	8	2(25%)	6(75%)	1.6[0.2,22.5]	
**Use of Antibiotic medicines after procedure**					0.3488

No (%)n	16	6 -38%	10 -62%	reference	
Yes (%)n	6	1 -17%	5 -83%	2.9[0.2,165.2]	
**Minimum time between procedures, days**					
Median[Min,Max]		10.5 [7,28]	42 [7,350]		0.0492
N	15	4	11		
**Maximum time between procedures, days**					0.0894
Median[Min,Max]		45.5 [28,112]	203 [14,875]		
N	15	4	11		
**Mean time between procedures, days**					0.0221
Median[Min,Max]		27.6 [20.1,28]	87.2 [14,350]		
N	15	4	11		
**Balloon use # of times**					0.7719
No (%)n	4	1(25%)	3(75%)	reference	
Yes (%)n	17	5(29%)	12(71%)	0.8[0.0,13.4]	
**Balloon (% of all procedures)**					0.269
Median[Min,Max]		100 [0,100]	50 [0,100]		
Mean ± SD		71.43+-48.8	57.92+-37.47		0.4819
N	22	7	15		

EIT - Endoscopic Incisional Therapy, PPI - Proton Pump Inhibitors, TEF - Tracheo-Esophageal Fistula

[illegible].
